# A time-motion study of community health workers delivering community-based primary health care in Neno District, Malawi

**DOI:** 10.1186/s12960-023-00839-z

**Published:** 2023-06-26

**Authors:** Moses Banda Aron, Myness Kasanda Ndambo, Fabien Munyaneza, Manuel Mulwafu, Henry Makungwa, Basimenye Nhlema, Emilia Connolly

**Affiliations:** 1 Partners In Health/Abwenzi Pa Za Umoyo, PO Box 56, Neno, Malawi; 2 Division of Pediatrics, University of Cincinnati College of Medicine, 3230 Eden Ave, Cincinnati, OH 45267 United States of America; 3 Division of Hospital Medicine, Cincinnati Children’s Hospital Medical Center, 3333 Burnet Ave, Cincinnati, OH 45529 United States of America

**Keywords:** Community health workers, Time-motion, Health conditions, Household visits, Community-based primary health care

## Abstract

**Introduction:**

Community health workers (CHWs) are vital resources in delivering community-based primary health care, especially in low-and-middle-income countries (LMIC). However, few studies have investigated detailed time and task assessments of CHW's work. We conducted a time-motion study to evaluate CHWs' time on health conditions and specific tasks in Neno District, Malawi.

**Methods:**

We conducted a descriptive quantitative study utilizing a time observation tracker to capture time spent by CHWs on focused health conditions and tasks performed during household visits. We observed 64 CHWs between 29 June and 20 August 2020. We computed counts and median to describe CHW distribution, visit type, and time spent per health condition and task. We utilized Mood’s median test to compare the median time spent at a household during monthly visits with the program design standard time. We used pairwise median test to test differences in median time duration for health conditions and assigned tasks.

**Results:**

We observed 660 CHW visits from 64 CHWs, with 95.2% (*n* = 628) of the visits as monthly household visits. The median time for a monthly household visit was 34 min, statistically less than the program design time of 60 min (*p* < 0.001). While the CHW program focused on eight disease areas, pretesting with the observation tool showed that CHWs were engaged in additional health areas like COVID-19. Of the 3043 health area touches by CHWs observed, COVID-19, tuberculosis, and non-communicable diseases (NCDs) had the highest touches (19.3%, 17.6%, and 16.6%, respectively). The median time spent on sexually transmitted infections (STIs) and NCDs was statistically higher than in other health areas (*p* < 0.05). Of 3813 tasks completed by CHWs, 1640 (43%) were on health education and promotion. A significant difference was observed in the median time spent on health education, promotion, and screening compared to other tasks (*p* < 0.05).

**Conclusion:**

This study demonstrates that CHWs spend the most time on health education, promotion, and screening per programmatic objectives but, overall, less time than program design. CHWs deliver care for a broader range of health conditions than the programmatic design indicates. Future studies should examine associations between time spent and quality of care delivery.

**Supplementary Information:**

The online version contains supplementary material available at 10.1186/s12960-023-00839-z.

## Introduction

Community health workers (CHWs) play a vital role in the provision of community-based primary health care (PHC), especially in low- and middle-income countries (LMIC) in the journey towards Universal Health Coverage (UHC) [1–3]. Many CHWs share similar characteristics, including a community-based work setting, role definition connecting to facility-based primary care, and demographic factors, regardless of geographical location [4]. Generally, CHWs do not have professional training but are locally trained in the context of the specific intervention and primary care system and are chosen from the community they serve [5]. Ample literature demonstrates that CHWs contribute positively to primary health care with improved health outcomes, especially in remote and poor communities [2, 6–15].

With various needs and contexts worldwide, there are no standardized roles or responsibilities for CHWs [16]. However, despite different scopes and tasks, it is critical that CHWs are strongly supported, trained, and monitored for high-quality care delivery [17]. Several factors have been shown to affect CHW performance, including the number of assigned households and geographic areas, program complexity, training, knowledge and skills [18, 19]. The complexity of tasks and high demand can lead to additional workload and requests for CHW’s services, resulting in poor quality of programmatic outputs [20].

While there is no literature on the scope and workload of specific programs, several studies in sub-Saharan Africa, including Malawi, demonstrate that an increased number of households assigned to the CHW results in underperformance, dissatisfaction, and poor retention [11, 21–29]. With the increased workload, studies have shown that CHWs may select tasks that they prefer to do, especially ones they do best and or require less effort [4, 30, 31]. Despite the known challenges intrinsic to the intermediary CHW role in the community, there has been little research into directly observed time measurements and scope of CHW activities compared to programmatic expectations.

Direct observation of health care workers, such as time-motion study (TMS), provides a high level of detail that can effectively be used for programmatic improvement and balancing workload [32]. In TMS, an external observer captures detailed quantitative data on the actions and duration required to accomplish a specific task, coupled with an analysis focused on improving efficiency [33]. Despite the usefulness of programmatic performance, there are few direct time-motion studies focused on measuring CHW activities [33–35]. From our literature review, CHW time-motion studies in India, South Africa, and Tanzania has shown that CHWs largely spend time on documentation and travel [2, 34, 35]. To our knowledge, only one study on CHWs' time utilization in Malawi focuses on cervical cancer and family planning [36]. To evaluate CHWs' current contributions to the health care system and identify opportunities for programmatic improvement around efficiency, quality, and effectiveness, we conducted a time-motion study of CHW activities in Neno District, Malawi. We aimed to evaluate how CHWs spend their time on home visits, disease focus areas, and specific tasks for iteration and improvement of CHW program scope, design, and overall primary care delivery.

## Methods

### Study and CHW program setting

Neno District is a rural and remote district in southwestern Malawi with an estimated population of 153,132 in 2023 [37]. Most people in Neno are subsistence farmers who live on less than 1.90 USD daily and have little access to electricity in the district [38]. Few paved roads connect health facilities in most of the mountainous districts with rugged terrain challenging facility access with substantial interruptions in patients' ability to access and direct care delivery during the rainy season [39]. CHWs are vital for community-based primary care delivery with patient support and linkage to facility-level care.

Partners in Health (PIH), known locally in Malawi as Abwenzi Pa Za Umoyo (APZU), has accompanied the Government of Malawi through the Ministry of Health since 2007 to utilize CHWs in the delivery of rural community-based primary health care in the Neno district [15, 21]. CHWs are assigned to entire households to support the broader health needs of families with eight specific focus areas of high disease burden or need– HIV, tuberculosis (TB), non-communicable diseases (NCDs), sexually transmitted infections, child health, malnutrition, family planning (among women of childbearing age), maternal and neonatal health [15].

Neno District has 1233 CHWs providing care to all households in the district through monthly screening visits and targeted follow-up visits. Each CHW is assigned to between 20 and 40 households depending on the population and geographical region within the district. The CHWs are given a volunteer monthly stipend (38 USD) for time and transport, provided training and ongoing education and support, and service delivery tools such as flip charts. CHWs work within the Ministry of Health’s community health structure and alongside Health Surveillance Assistants (HSAs), community health care workers based at facilities in Malawi [11, 26]. HSAs supervise community activities, village clinics, immunization, and household-level care but are significantly understaffed and overworked within the healthcare system [26]. Thus, the CHWs act as “foot soldiers” for the HSAs within the community at a household level, providing screening, case finding, and follow-up.

### Study design and sample size

**Fig. 1:**
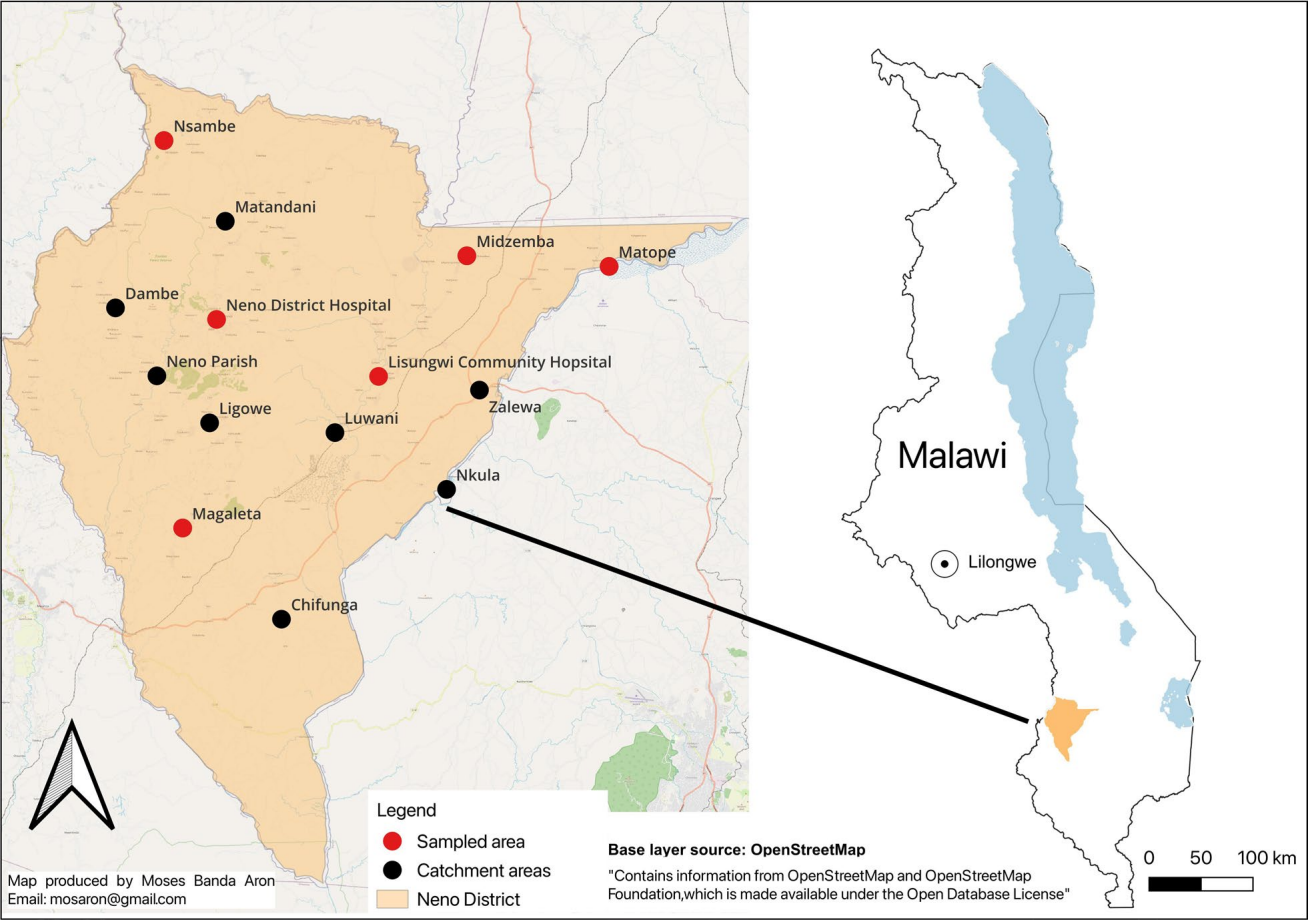
Map of Neno district showing the distribution of the catchment areas

We conducted a descriptive quantitative time-motion study utilizing a time observation tracker on CHWs in 6 of the 14 catchment areas in Neno District—Midzemba, Lisungwi, Matope, Nsambe, Magaleta, and Neno District Hospital (NDH). We selected these catchment areas due to diverse geography and representative populations. Midzemba, Lisungwi, and Matope are in a geographically flat area on the eastern side of the Neno district with easier terrain and transportation with large semi-urban populations. Conversely, Nsambe, Magaleta, and NDH catchment areas are located in mountainous, remote locations in the western part with rural, more secluded people (Fig. 1).

Sixty-four CHWs were proportionately sampled from each of the six catchment areas using the following formula:

$${n}_{h} =\frac{ {N}_{h}}{N} \times n.$$

${n}_{\mathrm{h}}$ is the CHW sample size for catchment area h, ${N}_{\mathrm{h}}$ is the CHW population size for catchment area h, *N* is the CHW total population size, and n is the total sample size, i.e. 64 CHWs. We further considered the CHW’s gender and distance to the health facility. To have the final CHW to be observed, we obtained the list of their names from the program team and used a random formula in Microsoft Excel to select.

### Data collection

We developed a time observation tracker focusing on several critical visits within the CHW program (Additional file 1). At the centre of CHW functions is a monthly household visit where CHW screens all household members within eight health condition focus areas depending on sex and age. Patients with known acute or chronic diseases or those who are pregnant are followed up with additional targeted visits. Through the program’s design, CHWs are expected to spend 45 to 60 min during a monthly household visit and less than 30 min for other visits. This study focused on monthly household visits with the recording of data for different visit types only when a CHW had a follow-up visit during the study observations.

The time observation tracker tool for time-motion study captured CHW’s demographics, including gender, age, education, catchment, the village where the CHW live and work, and the day and date when the observation was done. The time observation tracker captured times for the CHW starting and finishing work at the household, and specific tasks categorized type of visit and disease area of focus. The eight categories of visits were; monthly household visits, daily HIV patient visits, daily TB patient visits, referral follow-up visits, postnatal care (PNC) visits, escorting the patient to/from the facility, time at the health facility with the patient, and other assignments at the facility. The observation tool recorded the start and end times for each CHW task conducted at the household during visits, including check-in, documentation, health talks, screening, psychosocial support, and leading village-level meetings.

For the data collection, we hired four external data collectors without knowledge of the CHW program to limit data collection bias. We trained them for 3 days on the study objectives, the observation time tracker tool, and observation techniques. We pretested the data collection tool on four CHWs selected randomly from the NDH catchment area, with adjustments in the observation tool from these results before data collection. Additional health condition areas of COVID-19/respiratory diseases, malaria, and water sanitation and hygiene (WASH) were added to the observation tool eight disease focus areas based on initial observation pretesting. Each of the four data collectors observed 16 CHWs between 29 June and 20 August 2020. Each CHW was observed at least three times, covering a minimum of three households resulting in over 600 visits during data collection. Data collectors shadowed CHWs for the entire working day and recorded the start and end times of all the visits and tasks performed by recording the disease area covered in each task.

We notified CHWs in advance of data collection that they would be observed. However, they were not informed about the focus of observations or data collection to limit the influence of data collection on their routine work. We obtained consent from each CHW and the head of the household or household representative interacting with the CHW during the visit. We entered data into an Excel database from a paper-based observation tool and performed daily quality checks during data collection.

### Data analysis

The excel database was imported to R version 4.0.2 using Rstudio. We used descriptive statistics to characterize the community health worker’s demographic characteristics, tasks completed, and the health areas addressed. We utilized Mood’s median test to compare the median time spent at a household during monthly visits with the program design standard time. With a broad range of other types of visits, we only used data on the monthly household visits for further analysis of health areas and types of tasks. We grouped health conditions to track time into the following categories: (1) HIV, (2) TB, (3) non-communicable disease (NCDs), (4) COVID-19 and other respiratory diseases, (5) maternal and neonatal health, (6) family planning, (7) malnutrition for children under 5 years old, (8) child health for integrated management of childhood illnesses and immunizations, (9) sexually transmitted infections (STIs), (10) malaria and (11) other conditions. Other conditions included water sanitation and hygiene (WASH), malnutrition in persons over five years old, typhoid, and fever. We grouped the tasks completed during the household visit documented above into (i) health education and promotion, (ii) screening, (iii) documentation, (iv) psychosocial support, and (v) logistics. Logistics included patient check-in and check-out, introductions, medication side effect questions, and appointment reminders. We did not record the time it took for CHWs to travel from their house to their households. We used Mood’s median test post hoc test called the “Pairwise median test” to test differences in median time duration for health conditions and assigned tasks by producing box plots. In the boxplot output, the medians followed by a common letter, for example, “a” are not significantly different, and those with unique letters are significantly different from each other by the “pairwise median test” at a 95% confidence level of significance. Additional details regarding our study’s “Pairwise median tests” methods can be found in methodological studies published elsewhere [40–42].

## Results

### Demographics of CHWs

The 64 CHWs observed for this study represented 10.6% of the 601 total CHWs from these six sampled catchment areas. The proportion of CHWs observed from the total number of CHWs in each catchment area ranged from 7.3% to 12.1% (Table 1). Across all catchment areas, the CHW mean age was 37.8 years old, with estimated median households per CHW between 19.7 to 36.0 and 29 (45.3%) of the CHWs had primary-level education. There were slightly more female CHWs than males (65.5% vs. 34.5%), representing the total CHW workforce, where 66.9% are female (Table 1).

**Table 1:** Demographic characteristics of community health workers

Catchment area	Number of CHWs observed *n* (% of total)	CHW with primary education *n* (%)	CHW mean age (years)	CHW sex female *n* (%)	Estimated households per CHW in catchment^b^
Lisungwi	12 (11.5)	4 (33.3)	36.1	8 (66.7)	19.7
Magaleta	4 (7.3)	2 (50.0)	40.7	3 (75)	27.4
Matope	12 (10.4)	3 (25.0)	39.3	8 (66.7)	33.1
Midzemba	8 (8.6)	3 (37.5)	35.4	5 (62.5)	33.0
Neno DH^a^	16 (12.1)	10 (62.5)	39.6	10 (62.5)	36.0
Nsambe	12 (12.0)	6 (58.3)	36.5	8 (66.7)	35.1
Total	64 (10.6)	29 (45.3)	37.8	42 (65.6)	31.1

^a^District hospital

^b^Calculated from the total number of CHWs and number of households from monthly program reports in each catchment

### CHW visit type and time spent

We observed six hundred and sixty visits from the 64 CHWs, with an average of 10 visits during the study period. Most of the visits were monthly household visits (*n* = 628, 95.2%), with a median time of 34 min for the monthly household visits (Table 2). Of 628 monthly household visits observed, 147 (23.4%) were from the Neno District Hospital catchment area, with the least number of household visits from the Magareta catchment area (42, 6.7%) (Table 3). There was a considerable time range for monthly household visits, from 6 to 85 min. The median time of all monthly household visits (34.0 min, IQR 21.0) was statistically lower compared to the program estimates of 45–60 min (*p* < 0.001).

**Table 2:** CHW activities and time spent in minutes

Type of visit (*N* = 660)	Observed visits *n* (%)	Median time for the type of visit (min)
Monthly household visit	628 (95.2)	34
Daily HIV patient visit	7 (1.1)	28
Daily TB patient visit^a^	3 (0.5)	9
PNC visit^b^	13 (2.0)	43
Referral follow-up visit	8 (1.2)	7
Escorting patient to/from the facility	1 (0.2)	90^c^

^a^*TB* tuberculosis

^b^PNC postnatal care

^c^Time for one observation

**Table 3:** Monthly household visit time by the catchment area

Catchment area	HH visited *n* (%)	Median time (IQR)^b^	Time in minutes (Min.–Max.)
Lisungwi	111 (17.7)	37.0 (23.5)	14–85
Magareta	42 (6.7)	21.0 (12.5)	7–81
Matope	120 (19.1)	45.0 (15.0)	15–80
Midzemba	87 (13.9)	35.0 (22.5)	13–78
Neno DH^a^	147 (23.4)	25.5 (13.3)	6–84
Nsambe	121 (19.3)	33.0 (17.0)	15–81
Total	628 (100.0)	34.0 (21.0)^c^	6–85

^a^DH District hospital

^b^IQR Inter-quartile range

^c^t-test comparing to the program estimated time of 60 min = *p* < 0.001

### CHW time spent on health condition and task

There were 3043 touches on the tracked health conditions in the 628 observed monthly household visits. The highest number of touches for a health condition was 586 (19.3%) on COVID-19/respiratory disease, followed by 535 (17.6%) touches for TB and 506 (16.6%) for non-communicable diseases (NCDs). The fewest CHW touches were on malnutrition (*n* = 89, 2.9%) and child health (*n* = 48, 1.6%). CHWs spent the highest median time on STIs (9 min, IQR 7.0), followed by NCDs (6 min, IQR 9.0) (Table 4).

**Table 4:** Distribution of median time spent per health condition during the monthly CHW visit

Health condition (*N* = 3043)	Number of touches *n* (% of total)	Median time (interquartile range, IQR)
STI^a^	137 (4.5)	9 (7.0)
NCDs^b^	506 (16.6)	6 (9.0)
HIV	239 (7.9)	4 (6.0)
Maternal neonatal health (MNH)	141 (4.6)	4 (6.0)
TB^c^	535 (17.6)	4 (8.0)
Malaria	216 (7.1)	3.5 (9.0)
Child health	48 (1.6)	3 (3.0)
Family planning	147 (4.8)	3 (5.0)
COVID-19/respiratory diseases	586 (19.3)	3 (5.0)
Malnutrition	89 (2.9)	2 (3.0)
Other conditions^d^	399 (13.1)	1 (3.0)

^a^Sexually transmitted infections

^b^Non-communicable diseases

^c^Tuberculosis

^d^Includes water sanitation and hygiene, malnutrition for those over 5 years old, medication side effects, typhoid, and fever

Despite the greatest number of touches, COVID-19/respiratory diseases only had a median time of 3 min (IQR 5.0). However, the few touches corresponded with short median times of 3 (IQR 3.0) and 2 (IQR 3.0) minutes for child health and malnutrition.

**Fig. 2:**
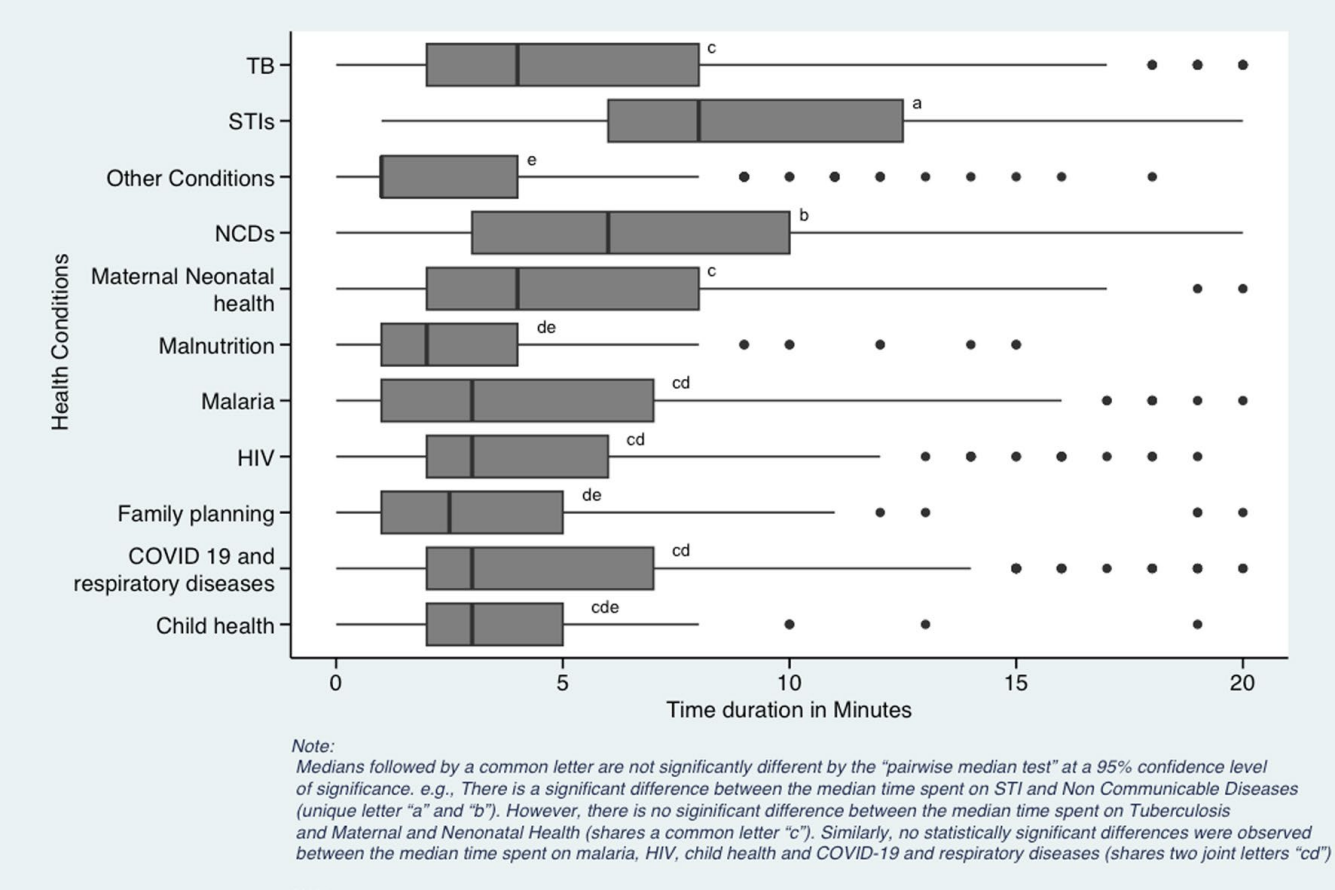
Comparison of median time spent per health condition during monthly CHW visit

In the pairwise comparison of median time spent on health conditions, we observed a statistically significant difference between the median time observed on STIs and NCDs (each has a unique letter, i.e. “a” and “b” indicating the difference in the median time spent on each condition), and a statistically significant difference between NCDs and the remainder of health conditions (*p* < 0.05) (Fig. 2). We observed no statistically significant difference between the median time spent on tuberculosis and maternal and neonatal health (shares a joint letter “c”). Similarly, no statistically significant differences were observed between the median time spent on malaria, HIV, child health and COVID-19 and respiratory diseases (shares two joint letters “cd”).

During the study period, CHW completed 3813 tasks in their monthly visits. Health education and promotion accounted for over 40% of all CHW tasks, with the longest median time of 6 min (IQR 8.0) (Table 5). Despite a high proportion of contacts with logistics, CHWs spent the shortest median time on this task at 1.0 min (IQR 1.0). Documentation and psychosocial support combined for 13% of all the tasks with the lowest median times at 2.0 min (IQR 3.0 and 2.0, respectively) (Table 5).

**Table 5:** Distribution of time spent per task during the monthly CHW visit

Tasks (*N* = 3813)	Number of times the task was completed *n* (% of total)	Median time (IQR)
Health education and promotion	1640 (43.0)	6.0 (8.0)
Screening	671 (17.6)	3.0 (5.0)
Documentation	442 (11.6)	2.0 (3.0)
Psychosocial support	83 (2.2)	2.0 (2.0)
Logistics^a^	977 (25.6)	1.0 (1.0)

^a^Logistics included appointment reminders and introductions at the household, but no travel time was recorded

**Fig. 3:**
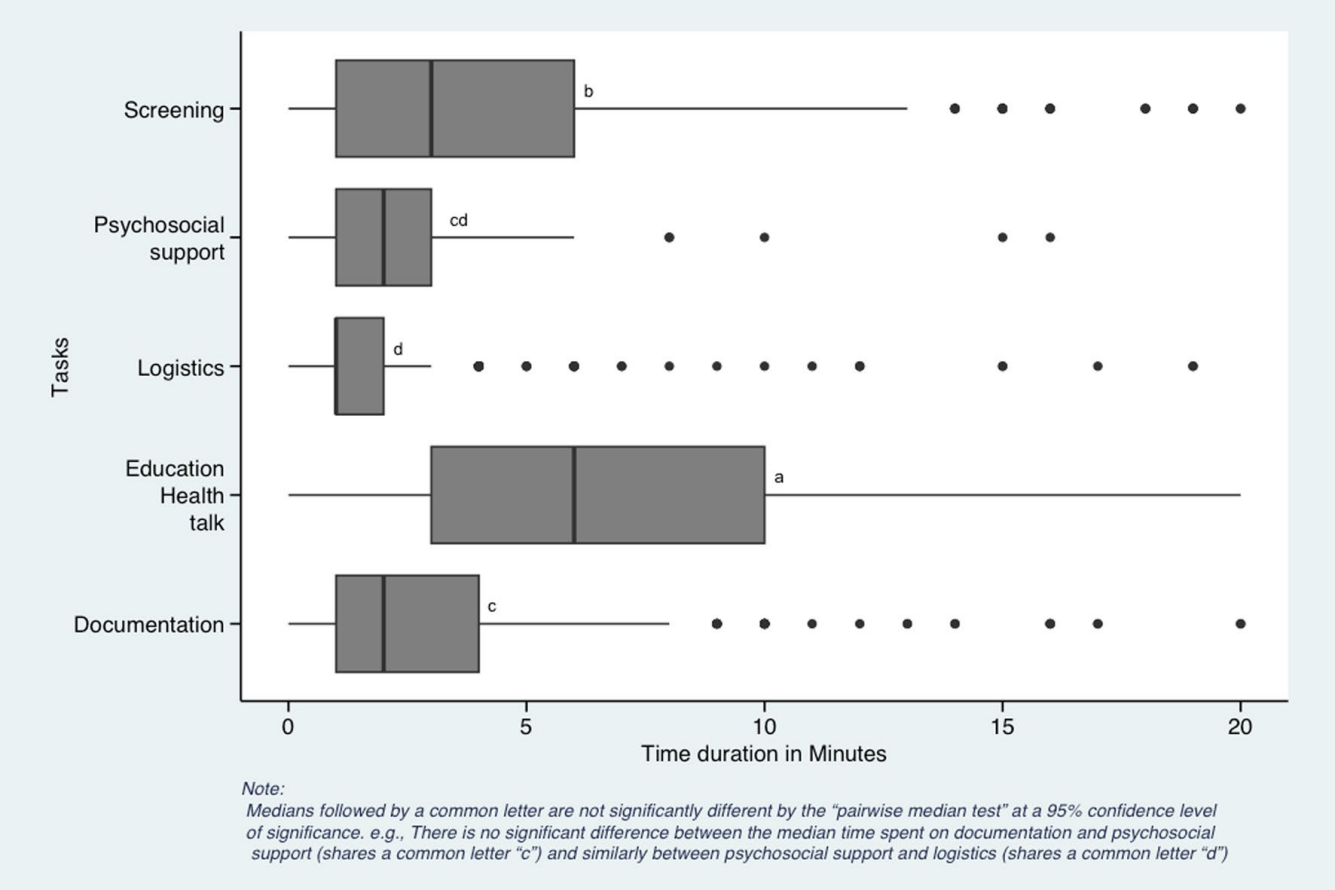
Comparison of median time spent per task during the monthly CHW visit

In comparing the median time spent per task during the CHW monthly home visits, there was a significant difference between health education and promotion and screening (each has a unique letter, i.e. “a” and “b”) with a higher median time compared to the time spent on other tasks (*p* < 0.05) (Fig. 3). We observed no significant difference between the median time spent on documentation and psychosocial support (shares a joint letter “c”) and similarly between psychosocial support and logistics (shares a joint letter “d”).

## Discussion

To our knowledge, only one study has reported on CHWs’ time utilization using direct time-motion observation in Malawi, focusing on cervical cancer and family planning [36]. However, our study focused on household visits and time taken for specific tasks among diverse multiple health areas. We found that CHWs in the Neno district spend significantly less time on monthly household visits than expected through program design, with a median time of 34 min. Despite our study showing that CHWs spend less time on monthly household visits than the expected 60 min from program design, prior studies done in Neno have shown that CHWs provide high-quality, patient-centred care with improved health outcomes in the community associated with CHWs work [15, 43–47].

We found that CHWs were spending > 50% of their time on disease screening and providing health education and promotion within the monthly household visit—a core design of the CHW program in Neno District [15]. Similar findings have been reported in India and South Africa where on average, CHWs spend 30 min on similar health education activities, nutrition screening, family planning, and immunizations [48, 49] as their core activities. However, a study done in Burkina Faso, Nigeria and Uganda found that CHWs spend longer visit times between 56 and 77 min on activities of testing and treatment components [50], which makes sense for the type of activities.

In our study, four out of six catchment areas have greater than 33 households assigned to each CHW despite this being within the expected number of households in the Neno district. While it is true that not all catchment areas with higher numbers of households demonstrated reduced median time spent on monthly visits in our study, it is important to consider that CHWs assigned to such areas may experience an increased workload and with less available time to spend on each individual household as shown in other studies [26, 51]. Further studies of our CHW program and others are required to further understand the ideal amount of time for high-quality care depending on visit type and expected tasks.

During monthly household visits, however, we found few CHW touches for STIs with significantly higher median times than all other health areas. This finding may be partly due to this study’s small number of observations. Still, it could also be partly due to the stigma and discomfort present with sexual-related health conditions in many communities with potentially a lack of health knowledge in the community [52]. Following STIs, the median time spent on NCDs was statistically higher than the remainder of all other health areas. With a correspondingly high proportion of touches, this finding is likely due to the growing burden of NCDs encompassing a complex, diverse set of health conditions in Malawi [53]. There is extensive literature on how CHWs have improved outcomes for NCD patients from the follow-up for missed facility visits to treatment outcomes [15, 54–56]. However, further studies are required to estimate how much time would be required for optimal disease screening, including NCDs, and ascertain the quality of the screening to pick up the disease.

Following STIs and NCDs, TB and COVID-19/respiratory disease had frequent touches by CHWs. This finding is likely due to the COVID-19 pandemic during the study period. In Neno, at the onset of COVID-19, CHWs were trained on preventive measures and COVID-19 education provision to their communities. Similar approaches have been reported by other LMICs as part of the COVID-19 response [57, 58]. While CHWs could play an important role in the fight against emerging diseases like COVID-19, there need to balance their workload, studies have shown that increasing the workload leads to burnout and poor performance [11, 18–20].

Another critical function of CHWs is to provide psychosocial support and more needed, especially in light of the COVID-19 pandemic-induced stress and anxiety [59–63]. However, we found that CHWs only spent a median of 2 min in each household, considered less than necessary to support individuals. In addition to eight health condition focus areas and COVID-19, CHW also focused on malaria and other conditions, including water sanitation and hygiene. This is unsurprising as the district is in southern Malawi, a region with a higher prevalence of Malaria (averaging 26% annually) [64]. Neno also has frequent typhoid and diarrheal disease outbreaks [65, 66]. This finding supports that these health areas are of concern within the community and should be given consideration for additions to the program through training and education tools.

On child health and malnutrition, we found that CHWs spent only a median of 3 and 2 min, respectively, with few touches for both health areas. This finding could be explained in part due to a “no-touch” policy introduced for COVID-19 infection control with a lack of personal protective equipment (PPE) for CHWs in Malawi. Similar “no-touch” policies were implemented for CHW programs during the Ebola outbreak in Guinea, Liberia and Sierra Leone, indicating the adoption of a syndromic surveillance approach [67, 68]. Thus, mid-upper arm circumference (MUAC) for malnutrition screening was replaced with the visual screening of the child’s weight, wasting, hair colour and texture [69]. These findings suggest that through community and health workforce engagement with active participation in shaping CHW tasks and focus areas, additional screening processes for all health areas with special attention to childhood malnutrition and child health should be adapted within the CHW program with the ongoing pandemic and future infectious disease outbreaks.

While CHWs appropriately only spend a short time (median of 1 min) on logistics such as appointment reminders, these reminders are crucial to the patients and improve health outcomes [70]. A systematic review of 25 studies found that informative appointment reminders through phone calls or text messages, or actual household visits improve facility attendance twofold [39]. In Neno District, we have observed the same with programmatic retentions of patients in care at 85% for HIV and 72% for NCDs driven primarily by CHW follow-up and appointment reminders [45, 46]. Further investigation of CHW patient support functions is needed to ensure that CHWs properly provide psychosocial care, education, and reminders to their households for facility follow-up.

The results of this study can help inform community health policy and human resource decision-making in Malawi. The National Community Health Strategy recommends one Health Surveillance Assistant (HSA) for every 1000 people to provide integrated care delivery of primary care at the catchment facility level [71]. It recommends that Community Health Volunteers (CHVs), aligned to the level and scope of the community health workers in this study, support the HSAs in service delivery but still needs to define the number of CHWs and scope of work. This study can provide essential information on the potential scope of work for CHVs and estimates of the time required for specific tasks and numbers of CHVs required at the household level to inform upcoming Community Health guidelines and policy. Further work in measuring an association between the amount of time and service done with further studies to compare time spent on tasks to service quality, such as individual patient follow-up is required to optimize community-based primary care delivery.

## Limitations

Our study has several limitations including limited generalizability with data from one rural district population in Malawi. However, we have cited numerous CHW programs in similar contexts and program designs worldwide in this study [2, 12, 30, 31, 34–36, 49, 56, 68] who could benefit from these findings on CHW time, health focus areas and time for specific tasks. Secondly, our sample size was small, which could influence our findings. However, we tried to minimize the effect of our CHW-limited sample size by a comprehensive approach to probabilistic selection criteria. This included considering geographic terrain, sex distribution of the CHW, and the CHW population within a specific catchment area relative to our sample; for example, the Magaleta catchment area has a smaller number of CHWs and finally, using a random function in Excel to generate the final list. We recommend conducting further studies that encompass a larger sample size of CHWs and are observed for more than two months with intervals within the year to compare potential seasonality or calendar changes in visits. Finally, with study observations during the onset of the COVID-19 pandemic, the CHW time spent in the household may have been affected due to fears of transmission and programmatic changes without the necessary PPE with adjustments in screening and time spent on specific health areas. However, these findings also provide valuable information on how CHW programs should adjust for incorporating COVID-19 and emerging outbreaks into essential health care.

## Conclusion

This study is the first time-motion study conducted in Malawi on CHW time utilization linked to specific health areas and tasks in service delivery. We found that CHW allocates the most time to health education, promotion, and screening activities, aligning with programmatic objectives. However, the overall time CHWs spend is less than what was initially planned or designed for the program.

The findings provide valuable insights for CHW program redesign and improvement. They highlight areas where adjustments in time allocation could enhance program effectiveness. The next steps include further investigation into the time spent on a visit, task or health area in connection to the quality of care provided with a direct linkage to CHW numbers and scope for optimized service delivery.

## Supplementary Information

**Additional file 1. **CHW Data collection observation tool.

## Data Availability

The datasets generated and/or analysed during the current study are available in the Zenodo repository, https://doi.org/10.5281/zenodo.6778792.

## Abbreviations

CHWs: Community health workers
PIH: Partners In Health
APZU: Abwenzi Pa Za Umoyo
TB: Tuberculosis
NCDs: Non-communicable diseases
S.T.I.s: Sexually transmitted infections
HIV: Human immunodeficiency virus
ART: Antiretroviral therapy
H.S.A.: Health Surveillance Assistant
PHC: Primary Health Care
LMIC: Lower and middle income countries
TMS: Time-motion study
PNC: Postnatal care
IQR: Inter-quartile range
NHSRC: National Health Science Research Committee
