# Supplementary material for: A time-motion study of community health workers delivering community-based primary health care in Neno District, Malawi
Source: Hum Resour Health. 2023 Jun 26;21:51. doi: 10.1186/s12960-023-00839-z (PMC10294529; doi:10.1186/s12960-023-00839-z)
Supplement: Supplementary file 1 — Additional file 1. CHW Data collection observation tool. [file 12960_2023_839_MOESM1_ESM.docx]

**Community health worker household activity record**

| Enumerator code: | | | | | | | |
| --- | --- | --- | --- | --- | --- | --- | --- |
| CHW ID: | | | | | | | |
| CHW Gender:                                 CHW Age:                                  CHW Education (circle):                                                                                                                 Primary level / Secondary level | | | | | | | |
| Catchment area/ Facility: | | | | | | | |
| Village: | | | | | | | |
| Date (DD/MM/YYYY): | | | | | | | |
| Day (circle): Sunday Monday Tuesday Wednesday Thursday Friday Saturday | | | | | | | |
| Household number: | | | | | | | |
| Time start work at household: __ __:__ __ | | | | Time stop work at household: __ __:__ __ | | | |
| **Category code** | **Task** | **Health condition** | **Location** | **Start time** | **End time** | **Duration** | **Comment** |
|  | **code** |  |  |  |  |  |  |
| Select category code from the list or specify other activity | Select task code from the list or specify other tasks | Select condition code from the list or specify other | 1 = Health Facility  2 = Assigned household  3 = community  4 = Travel  5 = Other (specify) | Enter activity start time | Enter activity end time | In minutes (use if start/end time not known) | Indicate notes on an observed category e.g. unable to time, categories combined etc. |
| 1 | \|____\| | \|____\| | \|__\| | __ __:__ __ | __ __:__ __ | \|__\|__\|__\| |  |
| 2 | \|____\| | \|____\| | \|__\| | __ __:__ __ | __ __:__ __ | \|__\|__\|__\| |  |
| … | \|____\| | \|____\| | \|__\| | __ __:__ __ | __ __:__ __ | \|__\|__\|__\| |  |
| n | \|____\| | \|____\| | \|__\| | __ __:__ __ | __ __:__ __ | \|__\|__\|__\| |  |

**Notes on general observations:**____________________________________________________________________________________________________________________________________________________________________________________________________________________________________________________________________________________________________________

| **Category code** | **Category** | **Health condition** | **condition code** |
| --- | --- | --- | --- |
| **Type of visit** | | **HIV** | A |
| 01 | Monthly household visit | **TB** | B |
| 02 | Daily HIV patient visit | **NCDs** |  |
| 03 | Daily TB patient visit | Mental Health | C1 |
| 04 | Referral follow-up visit | Diabetes | C2 |
| 05 | PNC visit | Hypertension | C3 |
|  | | Epilepsy | C4 |
| **Accompaniment** | | Asthma | C5 |
| 06 | Escorting patients to/from the facility | Other NCD | C6 |
| 07 | Time at a health facility with patient | **Maternal Health** |  |
| 08 | Other assignments at the facility (specify) | Screening Pregnancy | D1 |
|  | | ANC | D2 |
| **Task code** | **Task** | Facility delivery | D3 |
| **CHW Task conducted at Household/Community for Monthly household visits, patient daily visits, Referral follow-up visit, PNC visits and TRACE** | | Postnatal Care | D4 |
| a. | Check-in | Family Planning | D5 |
| b. | Documentation (filling referral forms, writing in the register, etc.) | **STIs** | E |
| c. | Education/Health talk | **Child Health** |  |
| d. | Screening | Malnutrition (under five) | F1 |
| e. | Psychosocial support | IMCI | F2 |
| f | Check out | Immunization (under five) | F3 |
| g. | Monthly Village level meeting | **Malaria** | G |
|  | | **WASH** | H |
| **CHW Task conducted at Household for Daily TB and HIV patient visit** | | Other (specify) | I |
|  |  |  |  |
| h. | Medication adherence (Assessment of whether the patient is taking medication as required) |  |  |
| i. | Medication side effects (Assessment of whether the patient is experiencing any side effects due to medication) |  |  |
| j. | Appointment reminder |  |  |
| k. | General psychosocial support |  |  |
|  | |  |  |
